# Neoadjuvant therapy in relation to lymphadenectomy and resection margins during surgery for oesophageal cancer

**DOI:** 10.1038/s41598-017-18879-6

**Published:** 2018-01-11

**Authors:** Joonas H. Kauppila, Karl Wahlin, Pernilla Lagergren, Jesper Lagergren

**Affiliations:** 1 Upper Gastrointestinal Surgery, Department of Molecular medicine and Surgery, Karolinska Institutet, Karolinska University Hospital, 17176 Stockholm, Sweden; 2 Cancer and Translational Medicine Research Unit, Medical Research Center, University of Oulu and Oulu University Hospital, 90014 Oulu, Finland; 3 Surgical Care Science, Department of Molecular medicine and Surgery, Karolinska Institutet, Karolinska University Hospital, 17176 Stockholm, Sweden; 4 Division of Cancer Studies, King’s College London and Guy’s and St Thomas’ NHS Foundation Trust, London, England

## Abstract

Differences in lymph node yield and tumour-involved resection margins comparing neoadjuvant therapy plus surgery with surgery alone for oesophageal cancer are unclear. Patients who underwent oesophageal cancer surgery in Sweden in 1987–2010 were included. Patients treated with neoadjuvant therapy were compared with those who underwent surgery alone. Outcomes were the number of examined lymph nodes (main outcome), number metastatic lymph nodes, and resection margin status. Rate ratios (RRs) and 95% CIs of lymph node yield were calculated by Poisson regression, and odds ratios (ORs) and 95% CIs of resection margin status by multivariable logistic regression, both adjusted for confounders. Among 1818 patients, 587 (32%) had received neoadjuvant therapy and 1231 (68%) had not. Lymph node yield was lower in the neoadjuvant therapy group (median 6 versus 8; adjusted RR 0.75, 0.73–0.78). Fewer metastatic nodes were identified following neoadjuvant therapy (median 0 versus 1; adjusted RR 0.76, 0.69–0.84). Neoadjuvant therapy associated to decreased risk of tumour-involved resection margins when adjusted for confounders except T-stage (OR 0.52, 0.38–0.70), but the association did not remain after adjustment for T-stage (OR 0.91, 0.64–1.29). Neoadjuvant therapy seems to decrease the lymph node yield and decrease the risk of tumour-involved resection margins by shrinking primary tumour.

## Introduction

The curatively intended treatment of most patients with oesophageal cancer includes oesophageal resection with lympadenectomy^1^. In the recent decade, the routine addition of neoadjuvant chemotherapy or chemoradiotherapy to surgery and centralization of surgery to fewer centres have improved the 5-year survival in oesophageal cancer^1,2^. More extensive lymphadenectomy has been associated with better survival in some studies^3,4^, but the independent prognostic role of more extensive lymphadenectomy has been challenged by recent studies controlling for confounding by surgeon volume^5,6^. The debate whether the differences in opportunities of lymphadenectomy between the main surgical approaches, i.e. open transthoracic, transhiatal, or minimally invasive oesophagectomy, influence the survival is also ongoing, but no clear survival differences have been shown thus far^7–10^. The use of neoadjuvant therapy might complicate the assessment of lymphadenectomy and how it may influence survival. By shrinking lymph nodes, it is possible that neoadjuvant therapy makes the removal or detection of nodes more difficult. The few investigations examining the association between neoadjuvant therapy and the number of resected lymph nodes show contradictory findings, with some studies reporting a reduction in the number of nodes, while other studies do not^11–17^. A main aim of neoadjuvant therapy is to shrink the primary tumour. This in turn could facilitate radical (R0) resection, but this question requires more research^18^.

Taken together, there is a need to better understand how neoadjuvant therapy influences the lymph node yield and resection margins status in oesophageal cancer patients who undergo surgery. The aim of this study was to clarify these questions while taking confounding into account, including the potentially critical factors surgeon volume and tumour stage.

## Methods

### Study design

In this population-based and nationwide Swedish cohort study from 1987 to 2010, the study exposure was the administration of neoadjuvant therapy (yes or no) before surgery for oesophageal cancer. The primary outcome was the number of removed and examined lymph nodes, while secondary outcomes were the number of removed and examined metastatic lymph nodes and the rate of radical resection without microscopic or macroscopic residual tumour in the resection margin (R0-resection).

### Cohort

Earlier versions of this cohort have been used for other clinical studies examining oesophageal cancer surgery^6,19,20^, and the updated current version has also been described elsewhere^21^. Briefly, the cohort included 98% of all oesophageal cancer patients who underwent curatively intended treatment in Sweden between 1987 and 2010. The patients were selected by combining data from the Swedish Cancer Registry for identifying all patients with oesophageal cancer, and the Swedish Patient Registry for selecting only patients who had undergone oesophagectomy. The information from these registers was linked for all individual patients using the Swedish personal identity number, a unique 10-digit identifier assigned to each Swedish resident upon birth or immigration, which is a well-validated tool for research purposes^22^. For collection of more detailed clinical data, including the number of nodes, resection margin status, surgeon volume and tumour stage, surgery charts and pathology records were retrieved from all hospitals conducting oesophageal cancer surgery in Sweden during the study period. The data retrieved from the medical records followed a detailed predefined protocol, an assessment that has been validated for high concordance^20^. Comorbidity data were retrieved from the Patient Registry and were defined and categorized using the most recently updated and well-validated Charlson Comorbidity Index^23^. The study was approved by The Regional Ethical Review Board in Stockholm, Sweden. All methods were carried out in accordance with relevant guidelines and regulations. Individual informed consent was not acquired as this is not necessary for this type of study (based on registry data and medical records) according to Swedish law.

### Treatment

The treatments, including neoadjuvant treatment, of the individual patients were agreed upon by surgeons and oncologists (typically in multidisciplinary meetings) together with the patients. The most frequently used neoadjuvant therapy was chemoradiotherapy, consisting of cisplatin- and fluorouracil-based chemotherapy supplemented by radiotherapy in 2Gy fractions for a total dose of up to 40Gy, but also radiotherapy and chemotherapy alone were used. Of those 1767 (97%) patients with data on surgical approach available, transthoracic resection with intrathoracic anastomosis was the dominating (96%) surgical procedure and a gastric tube which was pulled up and anastomosed to the proximal oesophagus was the preferred reconstruction. Only 4% of the patients underwent transhiatal oesophagectomy. No consensus on the extent of lymphadenectomy existed during the study period.

### Statistical analysis

All statistical analyses were carried out according to an *a priori* specified study protocol, defining and categorizing all exposures, outcomes and covariates. Because of the logarithmic distribution of the lymph node variables, Poisson regression was used to estimate ratios (RRs) with 95% confidence intervals (CIs) for associations between neoadjuvant therapy and number of removed and examined lymph nodes and metastatic lymph nodes. A stratified analysis was performed for T-stage groups. A subgroup analysis was conducted comparing patients receiving neoadjuvant chemoradiotherapy (excluding unknown neoadjuvant therapy and neoadjuvant chemotherapy or radiotherapy) to patients receiving surgery only. Multivariable logistic regression was used to calculate odds ratios (ORs) with 95% CIs for associations between neoadjuvant therapy and non-radical resection margins (R1/R2). In all models, the following covariates were selected as potential confounders: 1) age (continuous variable, per year), 2) calendar year (continuous variable), 3) tumour histology (adenocarcinoma or squamous cell carcinoma), 4) comorbidity (Charlson Comorbidity score 0, 1, or ≥2), 5) surgeon volume (0–6, 7–16, 17–46, or ≥47 cumulative number of oesophagectomies during the study period), and 6) pathological T-stage (T0-T1, T2, T3, T4, or Tx). Missing data covariate data were few (Table 1) and therefore handled by conducting a complete case analysis. The statistical software IBM SPSS v24.0 (IBM Corp., Armonk, NY) was used for all statistical analyses.

**Table 1:** Characteristics of 1818 patients who underwent surgery for oesophageal cancer with or without neoadjuvant therapy in Sweden in 1987–2010.

	Surgery alone	Neoadjuvant therapy and surgery	Total
	Number (%)	Number (%)	Number (%)
**Total**	1231 (68)	587 (32)	1818 (100)
**Time period**			
1987–1995	412 (34)	200 (33)	612 (34)
1995–2002	451 (37)	181 (29)	632 (35)
2003–2010	368 (30)	206 (36)	574 (32)
**Age (median, interquartile range)**	67 (60–73)	64 (58–70)	66 (59–72)
**Sex**			
Male	914 (74)	445 (76)	1359 (75)
Female	317 (26)	142 (24)	459 (25)
**Charlson’s Comorbidity Index**			
0	740 (60)	361 (62)	1101 (61)
1	305 (25)	139 (24)	444 (24)
≥2	186 (15)	87 (15)	273 (15)
**Type of resection**			
Transthoracic	1142 (93)	546 (93)	1688 (93)
Transhiatal	57 (5)	22 (4)	79 (4)
Missing	32 (3)	19 (3)	51 (3)
**Histology**			
Adenocarcinoma	594 (48)	197 (34)	791 (44)
Squamous cell carcinoma	634 (52)	389 (66)	1023 (56)
Missing	3 (0)	1 (0)	4 (0)
**Pathological T-stage**			
T0–1	211 (17)	211 (36)	420 (23)
T2	209 (17)	135 (23)	344 (19)
T3	534 (43)	149 (25)	683 (38)
T4	95 (8)	26 (4)	121 (7)
Tx	182 (15)	66 (11)	248 (14)
**Cumulative surgeon volume**			
Very low ( < 6)	336 (27)	153 (26)	489 (27)
Low (7–16)	245 (20)	150 (26)	395 (22)
Mid (17–46)	285 (23)	158 (27)	443 (24)
High (≥47)	328 (27)	105 (18)	433 (24)
Missing	37 (2)	21 (4)	58 (3)
**Number of removed nodes**			
Median (Interquartile range)	8 (5–16)	6 (3–12)	7 (4–15)
**Number of positive nodes**			
Median (Interquartile range)	1 (0–4)	0 (0–2)	1 (0–3)
**Resection margins**			
R0	816 (66)	456 (78)	1272 (70)
R1/R2	209 (17)	72 (12)	281 (15)
Missing	206 (17)	59 (10)	265 (15)
**Type of neoadjuvant therapy**			
Chemoradiotherapy	N/A	354 (60)	354 (60)
Radiotherapy	N/A	113 (19)	113 (19)
Chemotherapy	N/A	49 (8)	49 (8)
Missing	N/A	71 (12)	71 (12)

## Results

### Patient characteristics

Among 1821 patients included in the cohort, 1818 had information on neoadjuvant therapy and were selected for the present study. Of these, 587 (32%) had neoadjuvant therapy prior to surgery and 1231 (68%) had surgery alone. The regimen of neoadjuvant therapy was available for 516 patients, of which 354 (69%) received neoadjuvant chemoradiotherapy, 113 (22%) received neoadjuvant radiation therapy, and 49 (9%) received neaodjuvant chemotherapy. Characteristics of the study participants are presented in Table 1. The neoadjuvant therapy group contained younger patients and a larger proportion of patients with squamous cell carcinoma and tumours of favourable pathological tumour stage than patients in the surgery alone group, while comorbidity was equally distributed. The characteristics of the patients in the subgroup analysis of neoadjuvant chemoradiotherapy versus surgery were highly similar compared to the main analysis, and are shown in the Supplementary Table S1.

### Lymph node yield

**Figure 1:**
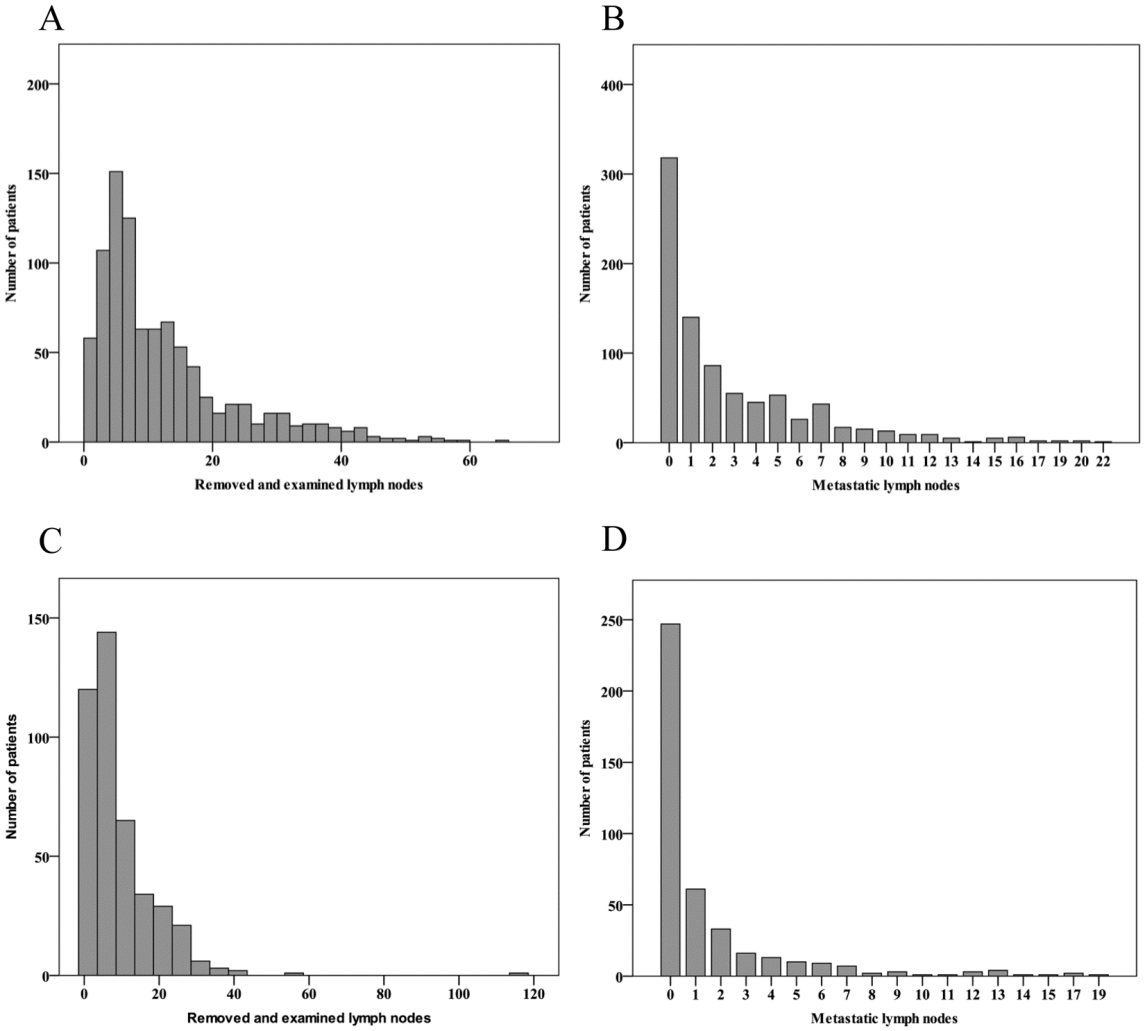
The distribution of number of removed and examined lymph nodes is shown for patients with surgery only (**A**) and patients undergoing neoadjuvant therapy before surgery (**C**). The distribution of number metastatic lymph nodes is shown for patients with surgery only (**B**) and patients undergoing neoadjuvant therapy before surgery (**D**).

Among all study participants, 1347 (74%) had information on the number of removed and examined lymph nodes and were thus included in this analysis. The distribution of number of removed and examined lymph nodes was skewed (Fig. 1). The number of nodes was lower among patients having had neoadjuvant therapy (median 6, interquartile range 3–12), compared to those having undergone surgery alone (median 8, interquartile range 5–16). The difference was confirmed in the fully adjusted Poisson regression for all pathological T-stages combined showing a 25% decrease in the number of lymph nodes after neoadjuvant therapy compared to surgery only (RR 0.75, 95% CI 0.73–0.78, and for each pathological T-stage analysed separately (Table 2). In the subgroup analysis of patients undergoing neoadjuvant chemoradiotherapy compared to those undergoing surgery, the difference was even more pronounced (32% reduction) in the fully adjusted Poisson regression model (RR 0.68, 95% CI 0.65–0.72, Table 2).

**Table 2:** Neoadjuvant therapy plus surgery compared to surgery alone for oesophageal cancer in relation to number of removed lymph nodes, expressed as rate ratios and 95% confidence intervals (CI).

Model	Patients Number	Surgery alone Rate ratio (95% CI)	Neoadjuvant therapy Rate ratio (95% CI)
Crude	1347	1.00 (reference)	0.77 (0.74–0.80)
Adjusted^a^	1307	1.00 (reference)	0.72 (0.69–0.75)
Adjusted^b^	1307	1.00 (reference)	0.75 (0.73–0.78)
**Stratified by T-stage** ^**a**^			
T0–1	319	1.00 (reference)	0.83 (0.77–0.89)
T2	296	1.00 (reference)	0.72 (0.66–0.77)
T3	578	1.00 (reference)	0.75 (0.71–0.80)
T4	92	1.00 (reference)	0.72 (0.60–0.87)
Tx	22	1.00 (reference)	0.48 (0.37–0.64)
**Subgroup analysis of patients with neoadjuvant chemoradiotherapy versus surgery**			
Crude	1184	1.00 (reference)	0.73 (0.70–0.76)
Adjusted^a^	1156	1.00 (reference)	0.65 (0.62–0.68)
Adjusted^b^	1156	1.00 (reference)	0.68 (0.65–0.72)
**Patients with neoadjuvant chemoradiotherapy versus surgery stratified by T-stage** ^**a**^			
T0–1	274	1.00 (reference)	0.82 (0.75–0.89)
T2	248	1.00 (reference)	0.62 (0.56–0.68)
T3	531	1.00 (reference)	0.68 (0.62–0.74)
T4	83	1.00 (reference)	0.48 (0.35–0.67)
Tx	20	1.00 (reference)	0.42 (0.30–0.58)

^a^Adjusted for age (per year), calendar year, Charlson’s Comorbidity Index, histology and surgeon volume.

^b^Adjusted for age (per year), calendar year, Charlson’s Comorbidity Index, histology, surgeon volume and *T-stage*.

The number of removed and examined metastatic lymph nodes was also lower among patients who had received neoadjuvant therapy (median 0, interquartile range 0–2), when compared to patients who had undergone surgery alone (median 1, interquartile range 0–4). This difference was confirmed in the fully adjusted model showing 24% decrease in number of metastatic lymph nodes after neoadjuvant therapy, compared to surgery only (RR 0.76, 95% CI 0.69–0.84, Table 3). The stratified analysis showed that the association was strongest in the pathological T-stage 3 category (Table 3). In the subgroup analysis of patients undergoing neoadjuvant chemoradiotherapy compared to those undergoing surgery, the difference was even more pronounced (32% reduction) in the fully adjusted Poisson regression model (RR 0.68, 95% CI 0.60–0.77, Table 3).

**Table 3:** Neoadjuvant therapy plus surgery compared to surgery alone for oesophageal cancer in relation to number of metastatic lymph nodes, expressed as rate ratios and 95% confidence intervals (CI).

Model	Patients Number	Surgery alone Rate ratio (95% CI)	Neoadjuvant therapy Rate ratio (95% CI)
Crude	1268	1.00 (reference)	0.53 (0.49–0.58)
Adjusted^a^	1228	1.00 (reference)	0.56 (0.51–0.61)
Adjusted^b^	1228	1.00 (reference)	0.76 (0.69–0.84)
**Stratified by T-stage** ^**a,c**^			
T0–1	290	1.00 (reference)	1.41 (1.04–1.91)
T2	279	1.00 (reference)	0.99 (0.82–1.19)
T3	549	1.00 (reference)	0.55 (0.48–0.63)
T4	88	1.00 (reference)	1.18 (0.89–1.57)
**Subgroup analysis of patients with neoadjuvant chemoradiotherapy versus surgery**			
Crude	1106	1.00 (reference)	0.44 (0.39–0.49)
Adjusted^a^	1078	1.00 (reference)	0.45 (0.40–0.51)
Adjusted^b^	1078	1.00 (reference)	0.68 (0.60–0.77)
**Patients with neoadjuvant chemoradiotherapy versus surgery stratified by T-stage** ^**a,c**^			
T0–1	245	1.00 (reference)	1.31 (0.93–1.84)
T2	232	1.00 (reference)	0.78 (0.61–0.99)
T3	502	1.00 (reference)	0.55 (0.46–0.66)
T4	79	1.00 (reference)	0.52 (0.29–0.92)

^a^Adjusted for age (per year), calendar year, Charlson’s Comorbidity Index, histology and surgeon volume.

^b^Adjusted for age (per year), calendar year, Charlson’s Comorbidity Index, histology, surgeon volume and T-stage.

^c^Estimates for Tx are not shown as they could not be calculated because of small numbers.

### Resection margins

A total of 1553 (85%) patients had data on resection margins and were included in the analysis of this outcome. The proportion of patients with tumour-involved resection margins (R1/R2) was lower (14%) in the neoadjuvant therapy group compared to surgery alone group (20%). After adjustment for confounders, except for T-stage, neoadjuvant therapy was associated with a decreased risk of tumour-involved resection margins (OR 0.52, 95% CI 0.38–0.70. When tumour T-stage was included in the fully adjusted model, no association remained (OR 0.91, 95% CI 0.64–1.29) (Table 4). Additionally, no associations between neoadjuvant therapy and resection margin status were found in the analysis stratified by T-stage categories (Table 4). In the subgroup analysis of patients undergoing neoadjuvant chemoradiotherapy compared to those undergoing surgery, the results were highly similar to the main analysis (Table 3).

**Table 4:** Neoadjuvant therapy plus surgery compared to surgery alone for oesophageal cancer in relation to positive resection margins, expressed as odds ratios and 95% confidence intervals (CI).

Model	Patients Number	Surgery alone Odds ratio (95% CI)	Neoadjuvant therapy Odds ratio (95% CI)
Crude	1553	1.00 (reference)	0.62 (0.46–0.83)
Adjusted^a^	1553	1.00 (reference)	0.52 (0.38–0.70)
Adjusted^b^	1553	1.00 (reference)	0.91 (0.64–1.29)
**Stratified by T-stage** ^**a,c**^			
T0–1	417	1.00 (reference)	1.90 (0.28–12.87)
T2	336	1.00 (reference)	1.03 (0.50–2.14)
T3	662	1.00 (reference)	0.95 (0.61–1.48)
T4	113	1.00 (reference)	0.47 (0.16–1.39)
**Subgroup analysis of patients with neoadjuvant chemoradiotherapy versus surgery**			
Crude	1343	1.00 (reference)	0.56 (0.39–0.81)
Adjusted^a^	1343	1.00 (reference)	0.50 (0.34–0.73)
Adjusted^b^	1343	1.00 (reference)	0.98 (0.63–1.54)
**Patients with neoadjuvant chemoradiotherapy versus surgery stratified by T-stage** ^**a,c**^			
T0–1	352	1.00 (reference)	1.60 (0.19–13.52)
T2	274	1.00 (reference)	0.62 (0.22–1.77)
T3	593	1.00 (reference)	1.07 (0.61–1.90)
T4	101	1.00 (reference)	1.71 (0.32–9.11)

^a^Adjusted for age (per year), calendar year, Charlson’s Comorbidity Index, histology and surgeon volume.

^b^Adjusted for age (per year), calendar year, Charlson’s Comorbidity Index, histology, surgeon volume and T-stage.

^c^Estimates for Tx are not shown as they could not be calculated because of small numbers.

## Discussion

The results of this study suggest that any neoadjuvant therapy, as well as neoadjuvant chemoradiotherapy, reduces the lymph node yield and reduces the proportion of tumour-involved resection margins in relation to tumour stage during surgery for oesophageal cancer. The latter association seems to be mediated by shrinkage of the primary tumour.

Among methodological strengths of the study are the population-based nationwide design, the complete and validated data collection, and the large sample size. To counteract confounding, which is an inherent source if bias in observation studies, adjustments were made for several key covariates. Lymph node yield increases with surgical experience^21^, and thus surgeon volume was adjusted for. Changes over time regarding surgical and oncological treatments and pathological examination of the surgical specimen were taken into account by adjusting for calendar year of the surgery. Because surgeons might prefer less radical surgery in frail patients, patient age and comorbidities were adjusted for. Surgeons operating on patients with more advanced tumours, including advanced tumours down-staged by neoadjuvant therapy, might conduct more radical surgery compared to less advanced tumours not receiving neoadjuvant therapy. Therefore, the results were adjusted for and stratified by T-stage. We did not have data on clinical T-stage, but could only assess pathological T-stage. In addition to the surgeon, the pathologist also has an important role in the final lymph node count^24^. Therefore, lymph node yield was labelled as removed and examined nodes. The smallest nodes in the specimen are harder to detect^25^, but yet up to 30–40% of the nodal metastases in gastroesophageal cancer are found in small (<5 mm) nodes^24,26,27^. This might affect the number of removed and examined lymph nodes in the present and previous studies evaluating the association between neoadjuvant therapy and lymph node yield. A weakness in the present study is the low median number of removed and examined lymph nodes, which might be considered a poor oncological resection. This might reflect the lack of centralization and consensus regarding the extent of lymphadenectomy in Sweden during most of the long study period. However, it is unlikely that the extent of lymphadenectomy would differ in those undergoing neoadjuvant therapy or surgery alone after adjustments for calendar year and surgeon volume.

The present study indicates that neoadjuvant therapy reduces the number of removed and examined lymph nodes. The available literature on this topic is limited. Randomized clinical trials have shown that neoadjuvant therapy increases the resectability of oesophageal cancer, but without reporting the lymph node yield^28–36^. Two post-hoc analyses of randomized clinical trials from the Netherlands (n = 320) and France (n = 195), and one French hospital-based cohort study of R0-resected patients (n = 536), suggested a reduced lymph node yield after neoadjuvant therapy compared to surgery alone for oesophageal cancer^11,16,17^. A register-based study from the United States on patients undergoing oesophagectomy for gastroesophageal cancers (n = 18,777) suggested that neoadjuvant therapy was associated with a decreased likelihood of obtaining 15 or more nodes, but did not assess specific numbers of nodes^37^. Another register-based study from the United States (n = 5,805), a Taiwanese register-based study (n = 2,151), and a hospital-based study (n = 111) from the United States found no association between neoadjuvant therapy and lymph node yield in oesophageal cancer^14,15,38^, but none of these studies adjusted the results for surgeon volume, or other potential confounders.

Neoadjuvant therapy was also associated with a decreased number of removed and examined metastatic lymph nodes in this study, which has also been observed earlier and is one of the goals of neoadjuvant therapy^11^. The stratified analysis showed that the association is the strongest in the high-T-stage category, suggesting that neoadjuvant therapy could reduce the number of lymph node metastases even when no significant down-staging occurs in the primary tumour.

The risk of tumour-involved resection margins was decreased in the neoadjuvant therapy group of the present study, which is in line with some previous studies^28–36,39^. The disappearance of the association after adjustment and stratification for T-stage suggests, in line with a previous study^40^, that the influence of neoadjuvant therapy on tumour-involved resection margins is mediated by shrinkage of the primary tumour.

This study has some potential clinical and research implications. The number of removed lymph nodes during surgery is considered an indicator of the quality of the esophagectomy^41^. This study suggests that the expected lymph node yield should be 25% lower after neoadjuvant therapy (and 32% lower after neoadjuvant chemoradiotherapy) than after surgery alone, even when adjusting for important confounders surgeon experience and time. This might not be a concern because recent large studies have not found any survival benefit of more extensive lymphadenectomy after neoadjuvant therapy^11^, or altogether in oesophageal cancer^5,6^. On the other hand, a recent large study from the Netherlands showed better survival (adjusted HR 0.77, 95% CI 0.68–0.86) after more extensive lymphadenectomy in oesophageal cancer after neoadjuvant chemoradiotherapy^42^. However, it is still unclear whether the previously suggested cut-offs for adequate lymph node yield are relevant in the neoadjuvant therapy era, or whether they are a proxy for skill and experience of the surgeon^6^. The association between neoadjuvant therapy and lymph node yield also indicates that future studies assessing lymphadenectomy should adjust for the use of neoadjuvant therapy.

In conclusion, this nationwide and population-based study with adjustment for several confounders indicates that neoadjuvant therapy reduces the number of removed and examined lymph nodes and the risk of tumour-involved resection margins in patients who undergo surgery for oesophageal cancer. These findings might contribute to changing the view regarding the need for a certain lymph node yield following neoadjuvant therapy and that neoadjuvant therapy should be taken into account in analyses of lymph node yield in future research.

## Electronic supplementary material

Supplementary Table S1
