# Supplementary material for: Neoadjuvant therapy in relation to lymphadenectomy and resection margins during surgery for oesophageal cancer
Source: Sci Rep. 2018 Jan 11;8:446. doi: 10.1038/s41598-017-18879-6 (PMC5765051; doi:10.1038/s41598-017-18879-6)
Supplement: Supplementary file 1 — Supplementary Table S1 [file 41598_2017_18879_MOESM1_ESM.pdf]

Supplementary data for: Neoadjuvant therapy in relation to lymphadenectomy and resection margins during surgery for oesophageal cancer

Joonas H Kauppila, MD, PhD, Karl Wahlin, MSc, PhD, Pernilla Lagergren, RN, PhD, Jesper Lagergren, MD, PhD

Content:

Supplementary Table S1. Characteristics of 1585 patients who underwent surgery for oesophageal cancer with or without neoadjuvant chemoradiotherapy in Sweden in 1987-2010.

Supplementary table 1. Characteristics of 1585 patients who underwent surgery for oesophageal cancer with or without neoadjuvant chemoradiotherapy in Sweden in 1987-2010.

	**Surgery alone**	**Neoadjuvant chemoradiation and surgery**	**Total**
	Number (%)	Number (%)	Number (%)
**Total**	1231 (78)	354 (22)	1585 (100)
**Time period**			
1987-1995	412 (34)	97 (27)	509 (32)
1995-2002	451 (37)	1112 (31)	563 (36)
2003-2010	368 (30)	145 (41)	513 (32)
**Age (median, interquartile range)**	67 (60 – 73)	64 (58 – 69)	66 (59 – 72)
**Sex**			
Male	914 (74)	271 (77)	1185 (75)
Female	317 (26)	83 (23)	400 (25)
**Charlson's Comorbidity Index**			
0	740 (60)	209 (59)	949 (60)
1	305 (25)	90 (25)	395 (25)
≥2	186 (15)	55 (16)	241 (15)
**Type of resection**			
Transthoracic	1142 (93)	338 (96)	1480 (93)
Transhiatal	57 (5)	15 (4)	72 (5)
Missing	32 (3)	1 (0)	33 (2)
**Histology**			
Adenocarcinoma	594 (48)	119 (34)	713 (45)
Squamous cell carcinoma	634 (52)	235 (66)	869 (55)
Missing	3 (0)	0 (0)	4 (0)
**Pathological T-stage**			
T0-1	211 (17)	146 (41)	357 (23)
T2	209 (17)	71 (25)	280 (18)
T3	534 (43)	79 (13)	613 (39)
T4	95 (8)	13 (12)	108 (7)
Tx	182 (15)	45 (20)	227 (14)
**Cumulative surgeon volume**			
Very low (<6)	336 (27)	71 (20)	407 (26)
Low (7 – 16)	245 (20)	91 (26)	336 (21)
Mid (17 – 46)	285 (23)	115 (32)	400 (25)

High (≥47)	328 (27)	75 (21)	403 (25)
Missing	37 (2)	2 (1)	39 (2)
**Number of removed nodes**			
Median (Interquartile range)	8 (5 – 16)	6 (3 – 12)	7 (4 – 15)
**Number of positive nodes**			
Median (Interquartile range)	1 (0 – 4)	0 (0 – 1)	1 (0 – 4)
**Resection margins**			
R0	816 (66)	278 (79)	1094 (69)
R1/R2	209 (17)	40 (11)	249 (16)
Missing	206 (17)	36 (10)	242 (15)
